# Supplementary material for: A ubiquitin-independent proteasome pathway controls activation of the CARD8 inflammasome
Source: J Biol Chem. 2022 May 14;298(7):102032. doi: 10.1016/j.jbc.2022.102032 (PMC9213247; doi:10.1016/j.jbc.2022.102032)
Supplement: Supplementary Figure S1 [file mmc1.pdf]

A ubiquitin-independent proteasome pathway controls activation of the CARD8 inflammasome

Jeffrey C. Hsiao, Atara R. Neugroschl, Ashley J. Chui, Cornelius Y. Taabuzing, Andrew R. Griswold, Qinghui Wang, Hsin-Che Huang, Elizabeth L. Orth-He, Daniel P. Ball, Giorgos Hiotis, & Daniel A. Bachovchin*

*Correspondence: bachovcd@mskcc.org

This PDF file includes:

Figures S1 and its Figure Legend

Figure S1

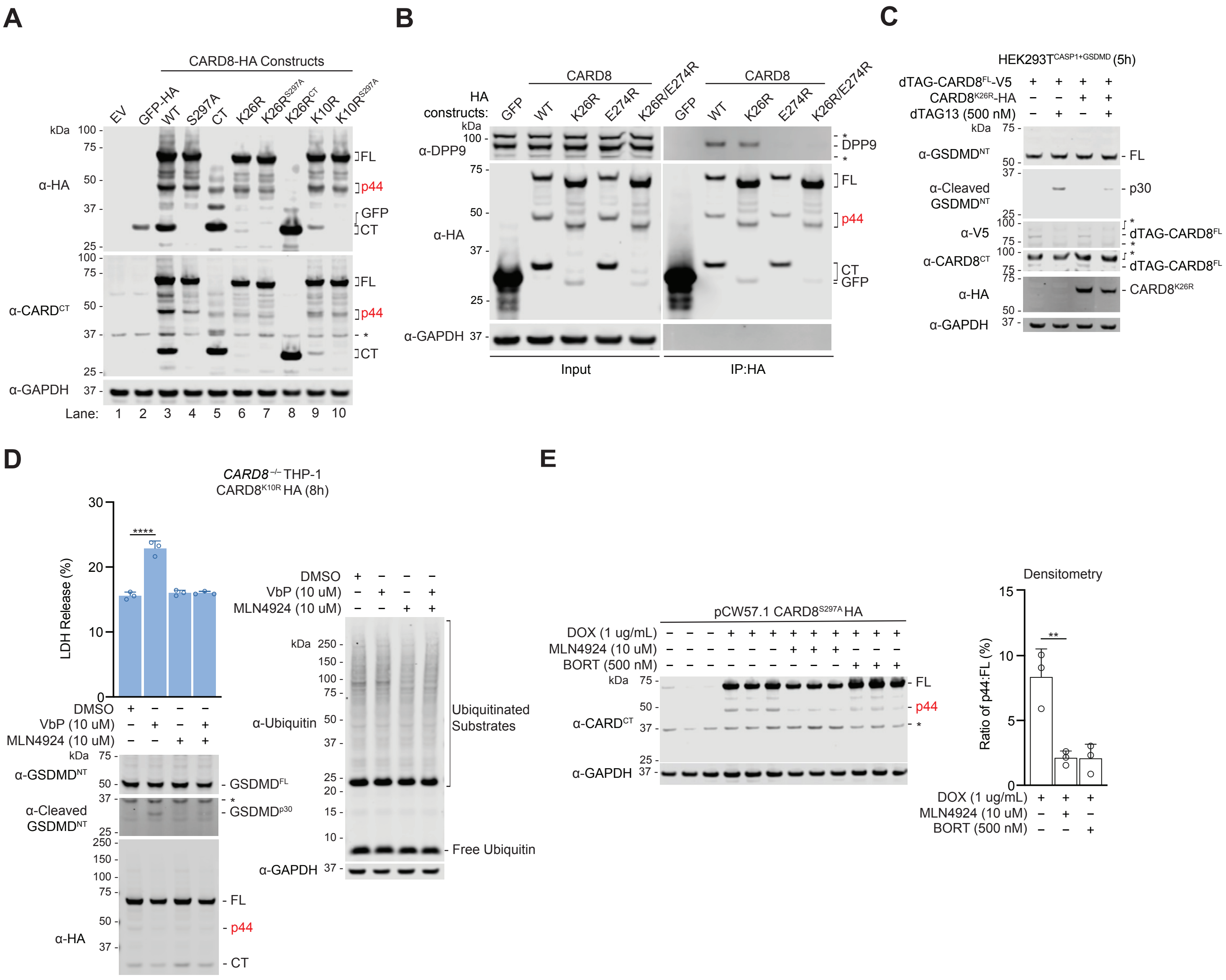

A
CARD8-HA Constructs
EV
GFP-HA
WT
S297A
CT
K26R
K26RS297A
K26RCT
K10R
K10RS297A
α-HA
α-CARDCT
α-GAPDH
FL
p44
GFP
CT
Lane: 1 2 3 4 5 6 7 8 9 10
B
HA constructs:
GFP
WT
K26R
E274R
K26R/E274R
CARD8
α-DPP9
α-HA
α-GAPDH
DPP9
FL
p44
CT
GFP
Input
IP:HA
C
HEK293TCASP1+GSDMD (5h)
dTAG-CARD8FL-V5
CARD8K26R-HA
dTAG13 (500 nM)
α-GSDMDNT
α-Cleaved GSDMDNT
α-V5
α-CARD8CT
α-HA
α-GAPDH
FL
p30
dTAG-CARD8FL
CARD8K26R
D
CARD8−/− THP-1
CARD8K10R HA (8h)
LDH Release (%)
****
DMSO
VbP (10 uM)
MLN4924 (10 uM)
α-GSDMDNT
α-Cleaved GSDMDNT
α-HA
GSDMDFL
GSDMDp30
FL
p44
CT
α-Ubiquitin
Ubiquitinated Substrates
Free Ubiquitin
α-GAPDH
E
pCW57.1 CARD8S297A HA
DOX (1 ug/mL)
MLN4924 (10 uM)
BORT (500 nM)
α-CARDCT
α-GAPDH
FL
p44
Densitometry
Ratio of p44:FL (%)
**

Figure S1. Characterization of CARD8 lysine mutants. (**A**) HEK 293T cells were transiently transfected with the indicated plasmid constructs prior to immunoblotting analyses. It should be noted that the laddering of the CT protein is likely due to incomplete dissociation of its oligomers by the sample preparation procedure for SDS-PAGE. (**B**) HEK 293T cells were transiently transfected with the indicated constructs. Lysates were harvested and HA-tagged proteins were enriched. The input and elution fractions were analyzed by immunoblotting. The CARD8 E274R mutation renders it unable to bind to DPP8/9. (**C**) HEK 293T$^{CASP1+GSDMD}$ cells were transfected with plasmids encoding the indicated plasmids. After 24 h, cells were treated with dTAG-13 for 5 h before immunoblotting analyses. (**D**) *CARD8*$^{-/-}$ THP-1 cells stably expressing CARD8^{FL} K10R were treated with the indicated compounds for 8 hours prior to LDH release and immunoblotting analyses. (**E**) A tet-on plasmid encoding CARD8^{FL} S297A was transfected into HEK 293T cells. After 20 h, cells were co-treated with the indicated drugs for 24 h before lysates were analyzed by immunoblotting. The densitometry ratio of CARD8^{p44} to CARD8^{FL} was quantified for each treatment group. Data in (**E**) (n=3) are means ± standard deviation (SD) of replicates. ** $p = 0.0085$ by Student's two-sided t-test. All immunoblots are representative of 3 or more independent experiments.
